# Physical Studies of P450–P450 Interactions: Predicting Quaternary Structures of P450 Complexes in Membranes from Their X-ray Crystal Structures

**DOI:** 10.3389/fphar.2017.00028

**Published:** 2017-01-30

**Authors:** James R. Reed, Wayne L. Backes

**Affiliations:** Department of Pharmacology and Experimental Therapeutics, Louisiana State University Health Sciences Center New OrleansLA, USA

**Keywords:** P450 cytochrome, protein–protein interaction (PPI), crystal structure, drug metabolism, microsomes, liver

## Abstract

Cytochrome P450 enzymes, which catalyze oxygenation reactions of both exogenous and endogenous chemicals, are membrane bound proteins that require interaction with their redox partners in order to function. Those responsible for drug and foreign compound metabolism are localized primarily in the endoplasmic reticulum of liver, lung, intestine, and other tissues. More recently, the potential for P450 enzymes to exist as supramolecular complexes has been shown by the demonstration of both homomeric and heteromeric complexes. The P450 units in these complexes are heterogeneous with respect to their distribution and function, and the interaction of different P450s can influence P450-specific metabolism. The goal of this review is to examine the evidence supporting the existence of physical complexes among P450 enzymes. Additionally, the review examines the crystal lattices of different P450 enzymes derived from X-ray diffraction data to make assumptions regarding possible quaternary structures in membranes and in turn, to predict how the quaternary structures could influence metabolism and explain the functional effects of specific P450–P450 interactions.

## Introduction

The mammalian cytochromes P450 (P450 or CYP) that are primarily housed in the endoplasmic reticulum are versatile catalysts that metabolize 75–90% of marketed drugs (Guengerich, 2006; Lynch and Price, 2007). These enzymes use a heme group, coordinated in one of the axial positions with a critical thiolate residue, to catalyze a diverse set of reactions through the mixed-function oxygenation of substrate carbons and heteroatoms (Guengerich, 2001). In addition to catalyzing a variety of reactions, the mammalian P450s also recognize a wide range of exogenous substrates due to a variety of closely related enzymes, representing three to four gene families, that have differing but overlapping substrate specificities (Nelson, 2006). Humans contain 57 P450 genes expressed in a tissue-specific fashion (Zanger and Schwab, 2013), with approximately 13 P450s being responsible for the majority of *in vivo* drug metabolism.

The P450 catalytic cycle relies on molecular oxygen and electrons supplied by the redox partners, cytochrome P450 reductase (CPR) and/or cytochrome b_5_. In this process, two distinct electron-transfer steps occur (White and Coon, 1980), each one following a protein-protein interaction with one of the potential redox partners (Miwa et al., 1979). Thus, protein-protein interactions are essential for optimal catalytic activities of the P450s. In addition to the interactions with redox partners, P450s also interact with each other. There is now considerable evidence that these P450–P450 interactions influence P450-mediated catalytic activities in form- and substrate-specific manners. In fact, studies now demonstrate that the function of a given P450 also can be altered by its own homomeric interaction (Davydov et al., 2010; Reed et al., 2012). Our recent review on this topic (Reed and Backes, 2016), in addition to several other excellent reviews (Davydov, 2011; Reed and Backes, 2012; Kandel and Lampe, 2014), have summarized the studies that demonstrated the effects of specific P450–P450 interactions on enzyme function and have categorized the effects mechanistically.

Although significant progress has been made to demonstrate that the interaction of specific P450s can influence enzyme function in membrane systems, very little is known about the specific quaternary structures of the P450 complexes in membranes and more importantly, into the ways in which different types of physical interactions between P450s influence function. Although many biophysical techniques have been employed to detect multimeric P450 in membranes, these studies, for the most part, are not conclusive with regards to specific protein-protein interactions within the P450 complexes or to the exact sizes of the multimeric complexes.

In contrast to the limits associated with the biophysical techniques used to analyze membrane systems, X-ray crystal structures provide clear images of protein conformation and intermolecular arrangement. In addition, these structures clearly elucidate the ways in which protein conformations change upon the binding of different substrates. The crystallographic analysis of human P450 forms has exploded this century. Numerous structures of mammalian P450s with a variety of substrates have been reported [reviewed in (Johnson and Stout, 2013)]. Because of limitations related to determining both the specific sizes of P450 complexes and the relative orientations of the P450 units contained in these heteromeric complexes, detailed information about the ways in which P450s may interact *in vivo* might be inferred from X-ray crystal structures and crystallographic lattices of the individual, purified forms. This approach was used previously (Hazai et al., 2005); however, when the study was published, there were only a limited number of available structures of the mammalian P450s to analyze. Thus, in the current review, the X-ray structures and the corresponding lattice arrangements of human CYP1A2 (PDB: 2HI4); rabbit CYP2B4 (PDB: 1SUO and 1PO5); human CYP2C8 (PDB: 1PQ2); human CYP2C9 (PDB: 1OG5); human CYP2C19 (PDB: 4GQS); human CYP2D6 (PDB: 2F9Q);; human CYP3A4 (PDB: 2V0M, 2J0D, 1TQN, and 1W0F) have been interpreted with respect to the reported effects of specific P450-P450 interactions on enzymatic function.

Unlike our previous reviews that emphasized the functional effects of P450–P450 interactions, this review will focus on the physical studies that have been used to elucidate the nature of P450 multimers and summarize the current state of knowledge derived from these studies regarding the physical characteristics of these oligomers. The crystal structures and crystallographic arrays of different P450s will then be analyzed for their possible relationships to protein–protein interactions in membranes and to the possible ways that these interactions may explain some of the functional effects associated with these interactions. Initially, we will discuss instances of homomeric P450–P450 interactions in membrane systems that appear to correspond very nicely with available crystal structures of these P450s. Various types of homomeric interactions identified in the crystal structures will then be used to propose explanations for the functional effects that are associated with specific heteromeric P450–P450 interactions. The possible role of protein conformational changes in the functional effects of P450–P450 interactions will also be addressed. Hopefully, the comparison of crystal structures will provide a framework for future studies to determine whether these functional effects can indeed be attributable to the 450–P450 interactions deduced from the crystallographic analyses.

## Physical Studies of Membrane Systems Containing P450s

### Determination of P450–P450 Binding Affinities

Purified P450s have a remarkable tendency to aggregate in aqueous solutions (Guengerich and Holladay, 1979; French et al., 1980; Dean and Gray, 1982; Myasoedova and Berndt, 1990; Myasoedova and Tsuprun, 1993). The arrangement of purified, detergent-free homomeric CYP1A2 (as well as CYP2B4) in solution was defined by electron microscopy as being hexameric with two layers of P450 trimers that are comprised of two types of distinct P450–P450 interactions (Tsuprun et al., 1986; Myasoedova and Tsuprun, 1993). Because of this characteristic of P450s, it is expected that the enzymes would have a very low *K*_D_ for homomeric interactions in membranes. Using a new approach to study P450–P450 interactions both physically and functionally, CYP2C9 was covalently immobilized to a self-assembled monolayer on gold film and its homomeric and heteromeric interactions with CYP2D6, CYP3A4, and CPR were monitored by surface plasmon resonance (Bostick et al., 2016). This technique allowed interactions between immobilized CYP2C9 and its binding partners in real time. From the data, the *K*_D_ constants for the various dimeric interactions were calculated and ranged from 1 to 18 nM. Similarly, in a global analysis of data derived from a thorough kinetic study involving CYP2E1-mediated metabolism of para-nitrophenol over a range of CYP2E1 and CPR concentrations, a *K*_D_ of 38 nM was approximated for the homomeric association of the CYP2E1 within a lipid milieu (Jamakhandi et al., 2007).

It should be pointed out that measurements of *K*_D_ which are based on volumetric concentrations, while providing a relative number that is more easily appreciated, are actually not relevant to P450–P450 binding affinities that occur in the two-dimensional lipid membrane (the interactions detected by surface plasmon resonance actually did occur with the enzymes in solution). Davydov et al. (2015) measured the two-dimensional binding affinities for both the homomeric and heteromeric interactions of CYP2E1, CYP3A4, and CYP3A5 by luminescence resonance energy transfer (LRET) after each of the pair of enzymes were variably labeled at cysteine residues with LRET molecular partners. The binding constants [defined as the P450 concentrations at which the fluorescence resonance energy transfer (FRET) level reached 50% of the maximum] for the various homomeric and heteromeric interactions ranged from 0.07 to 0.56 pmol/cm^2^, and the corresponding two-dimensional binding constant for homomeric CYP2E1 binding was 0.16 pmol/cm^2^ [as compared to a *K*_D_ of 38 nM (Jamakhandi et al., 2007)]. In many cases, the heteromeric P450 associations detected in these studies had higher binding affinities than the homomeric interactions. Because the surface density of P450 in the endoplasmic reticulum of mouse hepatocytes is estimated to be in the range of 0.6–2.8 pmol/cm^2^ (Watanabe et al., 1993), P450s are likely highly oligomerized *in vivo*.

### Studies Measuring Rotational Mobility of P450s

As summarized in our previous reviews (Reed and Backes, 2012; Brignac-Huber et al., 2016; Reed and Backes, 2016), the literature provides many different lines of evidence that P450s physically interact in lipid membranes by the application of a variety of techniques that studied the rotational mobility of P450s such as the following: (1) optical rotary diffusion (ORD) following photolysis of the ferrous P450-CO complex with plane polarized light (McIntosh et al., 1980; Gut et al., 1982, 1983; Kawato et al., 1982; Iwase et al., 1991); (2) electron paramagnetic resonance (EPR) of P450s containing cysteine groups that were labeled with a maleimide containing a spin probe (Schwarz et al., 1982); and (3) time-resolved polarized fluorescence of cysteine residues in P450 that were labeled with diiodofluorescein iodoacetamide (Greinert et al., 1982). Specifically, in these studies, the mobility and rotation of P450s incorporated in lipid bilayers of a given viscosity were compared with the theoretical mobility and rotation for a monomeric sphere with the approximate dimensions of the P450. These studies provided useful information regarding certain characteristics of the P450 complexes in the lipid environment. These parameters were also measured under conditions that would favor dissociation of P450 aggregates such as high lipid:P450 ratios (Kawato et al., 1982) or high salt (Iwase et al., 1991).

When microsomes were studied or when P450s were incorporated in artificial membranes at lipid:P450 ratios that are comparable to those in the endoplasmic reticulum at 37°C, there was a high proportion (approximately 50% or higher) of aggregated P450 in the membrane as indicated by immobilized protein over the time course of the experiments (Gut et al., 1982; Iwase et al., 1991). Lower temperatures favored higher proportions of immobilized P450s (Kawato et al., 1982). Although these data did not provide specific determinations of the sizes of the P450 aggregates, rotational relaxation/correlation times suggest that the mobile fraction of P450 in these microsomes and lipid bilayers is at least dimeric in size (and probably larger) (Kawato et al., 1982), which in turn, indicates that the immobilized fraction is represented by higher degrees of aggregation, and the fluorescent study generated data consistent with a rotating hexameric aggregate (Schwarz et al., 1982). Interestingly, the fluorescent study indicated that changes in the angle between orientation of the fluorescent label on CYP2B4 and the plane normal to the membrane occurred upon substrate binding and reduction by CPR. This was indicative of functional changes in protein conformation (and not protein aggregation) that could correspond to the change from the open to the closed conformation of CYP2B4 [as determined in X-ray crystal structures (Scott et al., 2003; Scott et al., 2004)].

The interaction of rabbit CYP1A2 and CYP2B4 is the most studied P450–P450 interaction. For many substrates, the functional effects of this interaction are stimulation of CYP1A2-mediated activity and inhibition of that by CYP2B4 (Backes et al., 1998; Reed et al., 2010). There is also evidence that CYP1A2 (and other P450s) can form homomeric complexes (Reed et al., 2012). Although rotational studies show that an excess concentration of either lipid or CPR can disperse homomeric aggregates of CYP1A (Iwase et al., 1991) and CYP2B4 (Gut et al., 1982) within membranes, this does not diminish the significance of the high-affinity heteromeric CYP1A2-CYP2B4 interaction *in vivo* (Backes et al., 1998; Cawley et al., 2001).

Many P450–P450 hetero-interactions have been shown to be resistant to disruption by CPR. The interaction of CYP2C9 with both CYP2D6 and CYP3A4 (which both result in inhibition of CYP2C9-mediated activity) was evidenced in the presence of equimolar or excess CPR (if the P450s were mixed before CPR was added) (Subramanian et al., 2009, 2010). The effect of CYP2E1 on the apparent *K*_m_ of CYP2B4 for metabolism of benzphetamine also was observed when CPR concentrations were in excess (Kenaan et al., 2013). Thus, some mixed P450 complexes are clearly refractory to dissociation upon the binding of CPR. Taking into account these findings and the fact that many heteromeric P450 interactions tend to display higher binding affinities (discussed above), these interactions may occur preferentially in biological membranes.

### Cross-Linking Studies with P450s

Another method that has been used to demonstrate P450 complex formation in membranes and to estimate the size(s) of P450 multimers is chemical cross-linking. There is some evidence to suggest that the hexameric level of aggregation for P450s in solution (Tsuprun et al., 1986; Myasoedova and Tsuprun, 1993) (discussed above) may also exist when the P450s are incorporated in membranes. Covalent crosslinking of CYP2B4, either in detergent-free solution or when incorporated into lipid vesicles of varying composition, generated virtually identical cross-linked protein bands in terms of mass distribution and densities (Myasoedova and Berndt, 1990). This suggests that the levels of aggregation were similar for CYP2B4 under either condition. This conclusion also was drawn from gel filtration studies with CYP2B1 showing that the aggregation state of the enzyme was unaffected by its association with dilauroylphosphatidylcholine (DLPC) (Rietjens et al., 1989).

Chemical cross-linking studies have shown that amine-reactive cross-linkers generate high levels of cross-linked P450s in incubations with purified, reconstituted systems (Reed et al., 2010) (Myasoedova and Berndt, 1990). The cross-linked proteins are characterized by a range of bands (dimeric to at least tetrameric) with tight grouping of three or more closely migrating bands for each size of oligomer when the samples are separated by PAGE indicating that multiple amine groups are in close proximity at the interface of the interacting P450s.

In contrast, studies of P450–P450 interactions using cysteine-reactive cross linkers have generated only a limited sized range of P450 multimers showing primarily dimeric (and in some cases trimeric) associations without significant evidence of higher levels of associations (McIntosh and Freedman, 1980; McIntosh et al., 1980; Davydov et al., 2015). Thus, cysteine–cysteine interactions between P450s may be more representative of specific, evolutionarily conserved interactions between the P450s. Certainly, this would seem to be the case for the homomeric interaction of CYP2C8 as disulfide bonds between the cysteines of adjacent protein units in CYP2C8 dimers helped stabilize the interaction when the enzyme was expressed in cells (discussed below).

Tsalkova et al. (2007) and Davydov et al. (2015) have designed a number of studies regarding protein interactions involving CYP3A4 that used site-directed mutagenesis to generate CYP3A4 mutants in which various cysteine residues in the enzyme were substituted for serine or alanine. In one study examining cysteine cross-linking with these mutants, the interaction of CYP3A4 was suggested to be dominantly trimeric both in solution and in membranes. Furthermore, the interaction apparently involved two different types of contact between the trimer units as indicated by the disappearance of the trimeric cross-linked band in gel electrophoresis (leaving the cross-linked dimer as the prevalent band) when one of the conserved cysteines was mutated to a serine (Davydov et al., 2015). The cross-linking experiment in this study also provided support for predictions of the arrangement of units in CYP3A4 oligomers that were based on examination of the X-ray crystal structure of the P450. Cysteine-reactive cross-linking was also used effectively with site-directed mutagenesis to demonstrate the dimerization of CYP2C8 and to elucidate details regarding the quaternary structure of the dimer (Hu et al., 2010). The details regarding the cross-linking studies involving CYP3A4 and CYP2C8 are discussed below in the section regarding the crystal structures of the P450s and their relationship to the homomeric P450–P450 interactions in membranes.

### Fluorescent Studies with P450s

Studies with fluorescently labeled [or P450s labeled with probes capable of fluorescence or luminescence resonance energy transfer (LRET)] P450s have also revealed aspects of P450–P450 interactions. Originally, Davydov et al. (2001) used different thiol-reactive fluorescent compounds to label the cysteine residues in P450s and CPR. FRET involving the variably labeled enzymes provided physical evidence for the interactions of CYP1A2 and CYP2B4 in addition to their interactions with CPR. This study provided physical evidence that the binding of CYP1A2 and CPR was enhanced when the P450 was in a mixed complex with CYP2B4, a finding that was predicted previously by kinetic analyses of CYP1A2- and CYP2B4-mediated metabolism as a function of the CPR concentration (Backes et al., 1998).

Luminescence resonance energy transfer and FRET experiments have been useful in demonstrating the homomeric interactions of CYP3A4 (Fernando et al., 2007; Davydov et al., 2013, 2015). FRET caused by the binding of 1-pyrenebutanol to the active site and its resulting interaction with the heme group of a CYP3A4 mutant (that did not display cooperative binding of the compound like the wild type enzyme) indicated a simple binding equilibrium (Fernando et al., 2007). However, spectral experiments using constant variation of substrate and enzyme concentrations (Job’s titration) indicated enzyme oligomerization influenced substrate binding. It was then shown that the binding of apo form of the mutant CYP3A4 to the holo-enzyme resulted in a loss of cooperative binding typically associated with the wild type enzyme, and removal of the apo form restored cooperativity.

Luminescence resonance energy transfer of appropriately labeled CYP3A4 CYS-depleted mutants (discussed above) was also used to demonstrate that homomeric interaction of the P450 possibly explained the heterotropic activation of CYP3A4-mediated metabolism by α-naphthoflavone (ANF) (Davydov et al., 2013). Interestingly, the effects of ANF and the oligomerization of the P450s were coincident with high surface density of the P450 in the proteoliposomes. The homomeric oligomerization of the enzyme was cooperative as shown by the LRET. Furthermore, using FRET to measure the interaction of a fluorescent substrate with pyrene-labeled CYP3A4 at different positions (in addition to the LRET technique), it was shown that ANF (and other substrates) could affect the degree of oligomerization of the enzyme at low protein concentrations by binding to a peripheral, allosteric site on the enzyme that was far removed from the active site (Tsalkova et al., 2007; Davydov et al., 2012, 2013; Sineva et al., 2013). The effects of ANF on enzyme oligomerization occurred over the same enzyme concentration range that resulted in cooperative metabolism of 7-benzyloxy-4-trifluoromethylcoumarin by CYP3A4. Thus, the heterotropic effects of ANF on CYP3A4-mediated metabolism may relate to its ability to influence the oligomerization of the P450 and may not be caused solely by altering substrate binding through interactions within the active site as proposed previously (Korzekwa et al., 1998; Shou et al., 2001; Houston and Galetin, 2005).

Luminescence resonance energy transfer was also used to characterize the nature of P450–P450 interactions involving CYP3A4, CYP3A5, and CYP2E1, and, as mentioned above, the two-dimensional binding dissociation constants for each binary combination were generated from these data (Davydov et al., 2015). These data also demonstrated that the CYP3A protein–protein interactions (but not those involving CYP2E1) were cooperative with Hill coefficients ranging from 2 to 3.7. As in the study with ANF, the cooperative homomeric interaction of the LRET-labeled CYP3A was associated with cooperative metabolism of 7-benzyloxyquinoline by CYP3A. The study also showed that the interaction of CYP2E1 with CYP3A resulted in both stimulation of the *k*_cat_ of CYP2E1-mediated metabolism of 7-methoxy-trifluoromethylcoumarin and a decrease in the 7-methoxy-trifluoromethylcoumarin binding affinity of CYP2E1. This LRET study also implicated the likely quaternary structure of the CYP3A4 multimer which is discussed in the section below regarding P450 crystal structures.

Fluorescence resonance energy transfer and bimolecular fluorescence complementation (BiFC) studies have been invaluable in demonstrating specific homo- and hetero-interactions of the enzymes of the P450 monooxygenase system in natural membranes and in discerning the relative tendencies of these labeled proteins to interact after co-expression in COS1 cells and HEK293 T cells (Szczesna-Skorupa et al., 2003; Ozalp et al., 2005). Briefly, FRET or BiFC pairs were co-expressed as fusion proteins by linking the cDNAs of complimentary fluorescent probes to the cDNA of the P450 system proteins. These studies showed the homomeric interaction of CYP2C2 as well as its interaction with the CPR. Although the interaction with CPR could also be detected for CYP2E1, the homomeric interaction of CYP2E1 was not detected in the cellular membranes. However, the heteromeric interaction of CYP2E1 and CYP2C2 was detected. The studies also were significant in showing the roles of specific protein domains in mediating the protein-protein interactions. The *N*-terminal, membrane-spanning domain was critical for CYP2C2 self-oligomerization, whereas the cytosolic domain (N-truncated form of the enzyme) was implicated in the interactions of CYP2C2 with both the CPR and CYP2E1. These findings again support the assumption that heteromeric P450–P450 interactions occur more readily than homomeric interactions.

### Positional Heterogeneity of P450–P450 Complexes

One of the most striking attributes of P450–P450 complexes is their stability over time. This trait is ultimately responsible for the functional effects of P450–P450 interactions and has been demonstrated by many studies. A *k*_off_ for the dissociation of CYP2B4 oligomers was calculated in the FRET study by Davydov et al. (2001) (discussed above) by measuring the rate of change in FRET after mixing two populations of CYP2B4 in which the sulfhydryl groups were labeled with different fluorescent probes. The very low value for *k*_off_ calculated in this experiment was 0.44 h^-1^. Interestingly, the quasi-irreversible nature of the heteromeric interactions of CYP2D6 and CYP2C9 also were demonstrated functionally by showing that the changes in metabolism caused by the interaction of the enzymes could be influenced by the order that P450s and CPR were added to the reconstituted system (Subramanian et al., 2009). Excess CPR could only attenuate inhibition of CYP2C9-mediated activity, caused by its interaction with CYP2D6, when CPR was added concurrently or before addition of the second P450 and not when the two P450s were added before the CPR.

“Positional heterogeneity” (or “persistent conformational heterogeneity”) of P450–P450 complexes are synonymous terms that have been used to describe the quasi-irreversible nature of P450–P450 complexes (Davydov, 2011; Davydov et al., 2015). Davydov, working with various researchers, has clearly demonstrated the significance of this trait using a variety of approaches. Initially, heterogeneity in the CYP2B4 homomeric complex was demonstrated by hyperbaric studies of the ferrous CYP2B4-CO complex (Davydov et al., 1995). This study indicated that a specific population of the CYP2B4 subunits in P450 multimers was susceptible to a pressure-induced conversion to P420. The different populations of CYP2B4 also could be distinguished by the equilibrium between low and high spin forms of the enzymes, a transition that occurs very rapidly unless the spin state of the enzyme is “frozen” through the quasi-irreversible interaction with other P450s. Evidence that these traits were associated with multimeric enzyme complexes was demonstrated by loss of enzyme heterogeneity when the P450 was treated with detergent. Given that P450 half-lives range from 4 to 37 h (Zhukov and Ingelman-Sundberg, 1999), quasi-irreversible P450–P450 interactions could have a profound influence on drug metabolism.

The interaction of CYP1A2 and CYP2B4 was initially inferred by its functional effects by Backes in the mid to late 1990s (Cawley et al., 1995; Backes et al., 1998). These studies showed that upon interaction of the two P450s, CYP2B4-mediated activity was inhibited, whereas that of CYP1A2 was dramatically activated. Davydov provided physical evidence for the formation of this complex using the hyperbaric technique (Davydov et al., 2000b). In this study, P450 stability as a function of pressure was compared for CYP1A2 and CYP2B4. Unlike CYP2B4, the ferrous CYP1A2-CO complex did not demonstrate a susceptibility to convert to P420 under increasing pressure. More importantly, when CYP1A2 and CYP2B4 were pre-incubated for 9 h (but not when measurements were made immediately after mixing the P450s), the barotropic instability of CYP2B4 disappeared and the peak absorption of the Soret band of the CYP2B4-CO complex shifted from 450 to 448 nm, similar to that of CYP1A2. Furthermore, there was a “steeper” dependence of spin state change to the change in pressure when the CYP2B4 was in a complex with CYP1A2 indicating greater hydration of the CYP2B4 active site as a result of its interaction with CYP1A2.

More recently, Davydov et al. (2016) conducted a hyperbaric study to demonstrate a pressure-induced conformation change in CYP3A4 that was either dual-labeled with a LRET donor/acceptor pair or an electron spin probe. It was determined that only approximately 15% of the CYP3A4 population adopted the “high pressure” conformation at ambient pressure. Furthermore, the allosteric activator, testosterone, caused a shift to this “high pressure” conformation when bound to the enzyme. By selective modification of the protein with fluorescent and nitroxide side-chain spin probes, the region of the CYP3A most affected by the conformation changes was defined as the regions involving the α-helices, A/A′, and the meander loop and was shown to correspond to open and closed states of the enzymes. The pressure-induced conformation change was consistent with the putative change in the oligomeric state of the enzyme associated with ANF and other allosteric activators (discussed above) indicating that the conformational state of the protein is intimately related to its state of aggregation.

Conformational heterogeneity of many P450s has also been explored by using CO-flash photolysis of the ferrous CO-P450 complex (Koley et al., 1994, 1995, 1996, 1997). Because the CO-complex that absorbs maximally at 450 nm is photo-sensitive, the kinetics of CO-rebinding to a P450 can be monitored on a rapid time scale by stopped flow spectrophotometry after subjecting the complex to a flash of white light. In the study with CYP3A4 (Koley et al., 1995), the experiments clearly demonstrated three populations of enzymes corresponding to three conformations of the enzyme. Furthermore, the equilibrium between the conformers was influenced by the substrate bound to the enzyme. These results are quite consistent with the findings regarding CYP3A4 oligomerization and heterogeneity by Davydov and co-workers (discussed above). Specifically, a trimer of the P450 was implicated in which there was a conformational equilibrium that was influenced by substrate/effector binding.

Positional heterogeneity of P450 multimers has also been demonstrated by the multiphasic, time-dependent chemical reduction of populations of CYP2B4 (Davydov et al., 1985) and of CYP3A4 (Davydov et al., 2005) by dithionite. Subsequent to measuring the chemical reduction of CYP3A4, heterogeneity in the CYP3A4 multimer was demonstrated by its time-dependent, enzymatic reduction using the soluble reductase domain of the bacterial P450, BM3 (Fernando et al., 2008). These studies showed the rate of reduction was related to the spin state of the enzyme. The low spin form was chemically reduced most rapidly; whereas, high spin form was more rapidly reduced enzymatically indicating this form preferentially interacted with the redox partner. These studies showed that the differences in reduction due to spin state were related to heterogeneity within the enzyme oligomer by using detergents to disperse the P450 multimers whereupon the reduction of P450 became monophasic with respect to time. The solubilized P450 was refractory to a substrate-induced spin state change. However, the study examining chemical reduction of CYP3A4 by dithionite also utilized nanodiscs to disperse the CYP3A4. Nanodiscs utilize phospholipid and a membrane scaffolding protein that allows for the stabilization of small, discoidal lipid-bilayer particles that are reported to contain monomeric enzyme when mixed at appropriate relative concentrations (Baas et al., 2004). This was significant because nanodisc-incorporated CYP3A4 was capable of a substrate-induced spin state change (Baas et al., 2004). More recent studies have indicated that CYP3A4 incorporated into nanodiscs with CPR also was catalytically active (Grinkova et al., 2010; Denisov et al., 2015). Thus, the monomeric state of the P450 was not necessarily inactive as might be concluded from the studies using detergents to disperse the P450.

## The Relevance of P450 Crystal Structures to P450–P450 Interactions in Membranes

Because of the lack of detail concerning the arrangement of P450s in their multimeric form, the crystal structures and lattices have been interpreted for their potential relevance to the functional effects of P450–P450 interactions. This approach also has been used in a previous study (Hazai et al., 2005). Of the four types of P450–P450 interactions that were evaluated in the prior study, it was concluded that none of the regions involved in binding for a given P450 dimer overlapped with the regions involved in other types of dimers. Thus, there was the potential for large aggregates to be built from the different types of dimers, and any given aggregate could be characterized by a variety of P450–P450 contacts.

The assumption in drawing inferences from P450 crystal structures is that each type of P450 has a tendency to arrange itself in characteristic associations that also may be observed in solution and in lipid membranes. As the crystal structures are generated from soluble P450 mutants that lack the *N*-terminal, membrane-spanning sequence and thus, are favorable to the formation of crystals, there is some uncertainty about the relevance of these structures to P450–P450 interactions within a lipid milieu. Speculation about the quaternary structure of mixed P450 complexes is even more problematic given that crystal structures for mixed P450 complexes do not exist. Despite the limitations of the approach, P450 crystal structures reveal some intriguing interactions that could explain some of the functional effects related to the physical interactions of different P450s.

In order to discuss the potential significance of arrangements in P450 crystal lattices, it is necessary to provide a basic description of the prominent structural features of the P450 protein. The enzymes are comprised of approximately 460 amino acids making up a series of α-helices, β-sheets, and interconnecting regions that are organized around a central heme prosthetic group. The drug-metabolizing P450s are tethered to the membrane by the insertion of a hydrophobic, N-terminal tail. The N-terminal region is connected to the structural core of the protein, known as the catalytic domain, via the polar linking region that contains several cationic amino acids and protrudes out the cytosolic side of the membrane.

It has been postulated that the polar linking region would be positioned to interact with the anionic charges of the phosphate groups in membrane lipids as a means to modulate membrane positioning of the P450s (Ozalp et al., 2006) and presumably, the interaction with redox partners and other P450s. Both the membrane penetration and α-helical content of CYP1A2 were increased in the presence of anionic phospholipids (Ahn et al., 1998), and these changes may be attributable to stronger electrostatic interactions between the negatively charged phospholipids and the positively charged, polar linking regions of this P450. Furthermore, these protein-lipid interactions may allow for tighter packing of the polar linking regions between adjacent P450s as they might minimize the repulsion between the like-charged, cationic amino acids in the polar linker regions of adjacent enzymes. The cross-linking results with amine-reactive cross-linkers (discussed above) would be consistent with covalent bonds involving this region in adjacent protein units of P450 multimers. Interactions involving this region also have been implicated in a membrane-bound, dimeric CYP2C8 complex (discussed below). Interestingly, electrostatic interactions have been implicated in the homomeric interaction of CYP1A2 that causes inhibition of the enzyme (Reed et al., 2012) and have been shown to be involved in both the heteromeric CYP1A2-CYP2B4 (Kelley et al., 2005) and CYP1A2-CYP2E1 (Kelley et al., 2006) interactions. Future work is needed to show whether the electrostatic interactions between lipids and the polar linking regions are those responsible for these P450–P450 interactions.

P450s are wedge-shaped with two prominent sides that have important functional roles and are identified by their relative orientation with respect to the heme group. The distal side (relative to the heme group) protrudes out from the heme and contains the F and G helices that form the outer wall of the substrate binding cavity. For many P450s, the B-C loop and F-G loop/minor helices region between the F and G helices has been shown to play a role in substrate recognition (Ibeanu et al., 1996; Williams et al., 2004; Pochapsky et al., 2010). Studies examining P450s in nanodiscs indicate that the monomeric enzyme is tilted at an angle with respect to the membrane surface with the distal region partially buried in the membrane along with the hydrophobic, N-terminal tail of the P450 (Baylon et al., 2013). Indeed, truncated P450s lacking the N-terminal tails interact with membranes by virtue of the F-G region (Pernecky et al., 1993; Pernecky and Coon, 1996; Williams et al., 2000a; Scott et al., 2001). P450s demonstrate conformational flexibility, and dramatically different open and closed structures involving primarily changes in the positions of the B-C loop and the F-G helices have been identified for some P450s (Scott et al., 2003; Ekroos and Sjogren, 2006; Roberts et al., 2010).

The other prominent face of the P450 is the proximal side of the enzyme. This surface constitutes a broad, flat area with multiple, positively charged residues that facilitate interaction with and electron transfer from the redox partners, CPR and cytochrome b_5_ (Tamburini et al., 1986; Bridges et al., 1998; Sevrioukova et al., 1999; Davydov et al., 2000a). The results with amine-reactive cross-linkers may also implicate this region as the interface of P450 multimers. In the section below regarding P450 crystal structures, proximal-to-proximal interactions are observable in the crystal lattices of some P450s and may explain some of the functional effects attributable to P450–P450 interactions.

When considering the potential functional consequences of a protein–protein interaction using the crystal structure as a model, it is important to only consider crystal structure interactions in which the N-terminal ends of the interacting P450s can be contained in a single plane (representative of the surface of the membrane lipid bilayer) that does not intersect the center of the interface of the interacting units or have the two (or more) proteins projecting outward from opposite sides of the imaginary plane. Structures not meeting these criteria would not likely be plausible *in vivo* (unless P450s could interact on the opposing folds of the endoplasmic reticulum membrane) given the physical constraint that the P450s are known to be tethered to the membrane by the N-terminal tails.

Quaternary structures that have been elucidated from the crystal structures in this study have also been evaluated using the program, PDBePISA (Proteins, Interfaces, Structures, and Assemblies) (European Bioinformatics Institute, Hinxton, England). This algorithm evaluates the interfaces of protein–protein contacts within the crystal to assess their potential stability within the membrane and also predicts the sizes of complexes that would be formed from these intermolecular interactions. The strength of the interaction is evaluated based on the number of potential hydrogen bonds, salt bridges, and disulfide bonds that can be detected within the interaction and is quantified by a Complex Significance Score (CSS) that ranges from 0 to 1 (with 1 being a strong interaction). The analysis can also determine the interface area of the contact and the number of interacting residues between protein units for any given arrangement in the crystal.

**FIGURE 1:**
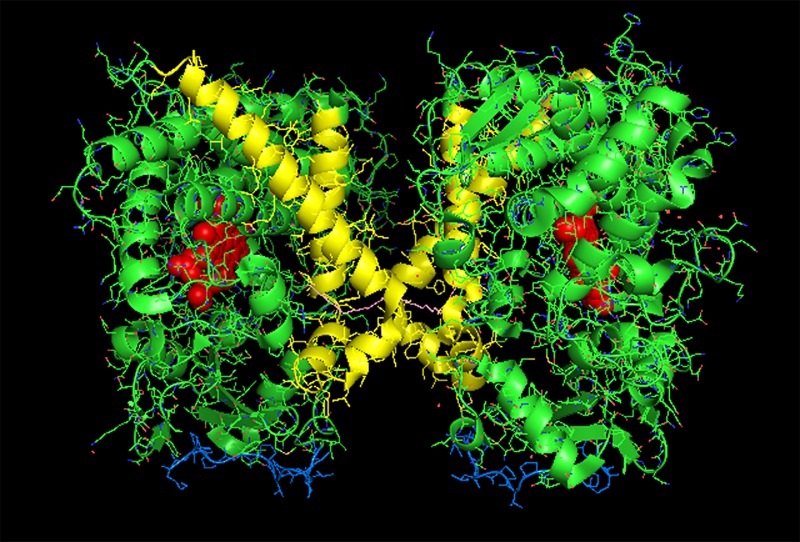
**Crystal structure of dimeric form of CYP2C8 bound to palmitate (PDB: 1PQ2).** The units of the dimer are oriented with the N-terminal region (shown in blue) at the base of the dimers in a “membrane plane” running perpendicular to the plane of the image. The heme groups are shown as red spheres with α-helices drawn as cylindrical features. The dimers are joined at F-G loop region. The F-G helices on the surface of the distal faces of the dimer are shown in yellow. Two molecules of palmitate bound on the periphery of the active site at the junction of the dimer are shown in pink. All molecular structures in this study were generated in PyMOL v. 1.8.4.1 (Schrṏdinger, Cambridge, MA, USA).

**FIGURE 2:**
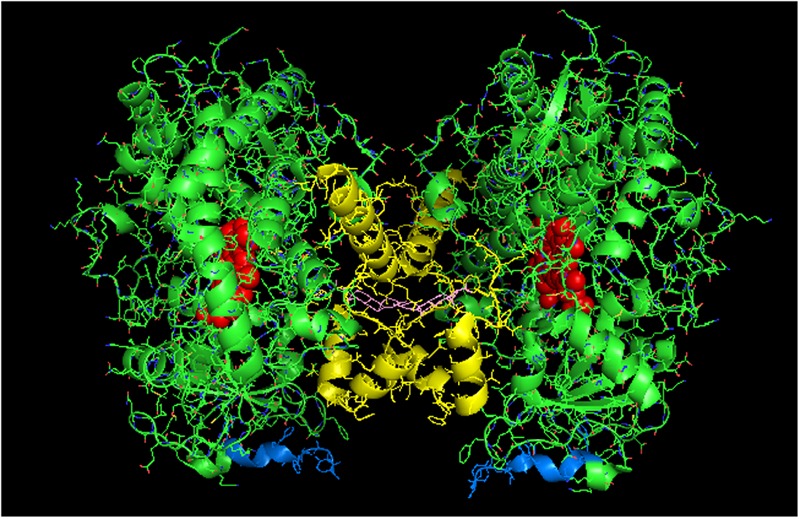
**Crystal structure of distal-to-distal dimeric form of CYP3A4 bound to progesterone (PDB: 1W0F).** The units of the dimer are oriented with the N-terminal region (shown in blue) at the base of the dimers in a “membrane plane” running perpendicular to the plane of the image. The heme groups are shown as red spheres with α-helices drawn as cylindrical features. The F-G helices region indicating the distal surface is shown in yellow. The peripheral effector site for binding of two molecules of progesterone are shown in pink.

Interestingly, evaluations of P450–P450 interactions using this program generally predict that the proteins exist as monomers (CSS scores close to 0) despite the preponderance of evidence for multimeric P450s in membranes (as described above). In conjunction with this trend, none of the P450–P450 complexes we evaluated in this study were predicted to be stable in the membrane (shown in **Table 1**) even though the direct evidence suggests that quaternary structures very similar to those identified from the CYP2C8 (**Figure 1**) and CYP3A4 (**Figure 2**) crystal structures have been demonstrated in membranes (as discussed below). The uncertainties regarding the *in silico* interpretation of intermolecular interactions may reflect the importance of the N-terminal, hydrophobic tails (which are absent in x-ray crystal structures of P450s) in stabilizing P450–P450 complexes in the membrane.

**Table 1:** Binding characteristics of the different homomeric complexes of P450 shown in the figures as determined from PISA analysis.

Complex Figure #	Nature of complex	*N*_res_^a^	Interface area (Å^2^)	Δ^i^G^b^ kcal/mol	*N*_HB_^c^	*N*_SB_^d^	*N*_DS_^e^	CSS^f^
CYP2C8 **Figure 1**	Distal-to-distal	11	265.6	-2.2	4	2	0	0.000
CYP3A4 **Figure 2**	Distal-to-distal	17	444.9	-6.4	0	0	0	0.000
CYP1A2 **Figure 3**	Proximal-to-proximal	17	523.6	1.3	0	0	0	0.000
CYP2B4 (closed form) **Figure 4** Interface 1^g^	Side-to-side	26	644	0.4	4	2	0	0.000
CYP2B4 (closed form) **Figure 4** Interface 2^g^	Side-to-side	25	789	-4.5	1	0	0	0.000
CYP2B4 (open form) **Figure 5**		18	290	2.6	4	2	0	0.000
CYP3A4 (open form) **Figure 6**	Proximal-to-proximal	20	469	-1.1	4	0	0	0.000
CYP2C9 **Figure 7**	Proximal-to-side	21	553.1	3.8	5	6	0	0.000
CYP2C19 **Figure 8**	Side-to-side	11	359.7	-4.4	3	0	0	0.047
CYP2C9 **Figure 9**^h^	Hexameric	17	398.3	-1.2	4	1	0	0.000

**FIGURE 3:**
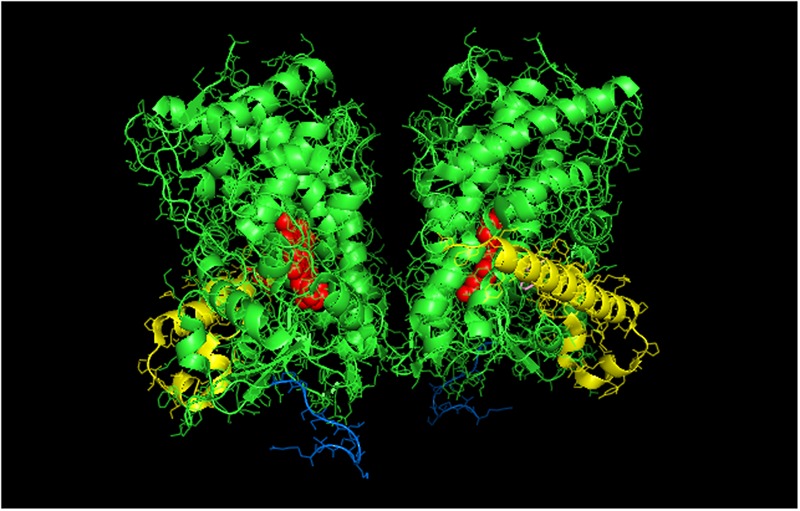
**Crystal structure showing lattice arrangement of proximal-to-proximal dimer of CYP1A2 bound to the inhibitor, ANF (PDB:2HI4).** The units of the dimer are oriented with the N-terminal region (shown in blue) at the base of the dimers in a “membrane plane” running perpendicular to the plane of the image. The heme groups are shown as red spheres with α-helices drawn as cylindrical features. The bound ligand, ANF is shown in the active site in a pink color. The F-G helices region on the distal face of the enzyme is shown in yellow.

**FIGURE 4:**
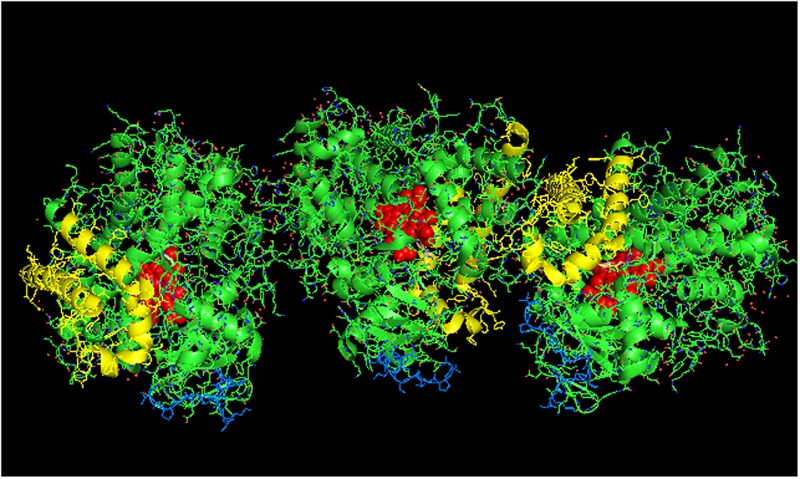
**Crystal structure showing side-to-side lattice arrangement of a CYP2B4 trimer of closed form CYP2B4 with 4-(4-chlorophenyl)imidazole (shown in pink) bound to the active site (PDB:1SUO).** The units of the trimer are arranged with the N-terminal ends (shown in blue) at the base of the units in a “membrane plane” running perpendicular to the plane of the page. Regions involved in the interactions include the G-, H-, J-, C-, and D-helices. The heme groups are shown as red spheres with α-helices drawn as cylindrical features. The F-G helices region on the distal face of the enzyme are shown in yellow.

**FIGURE 5:**
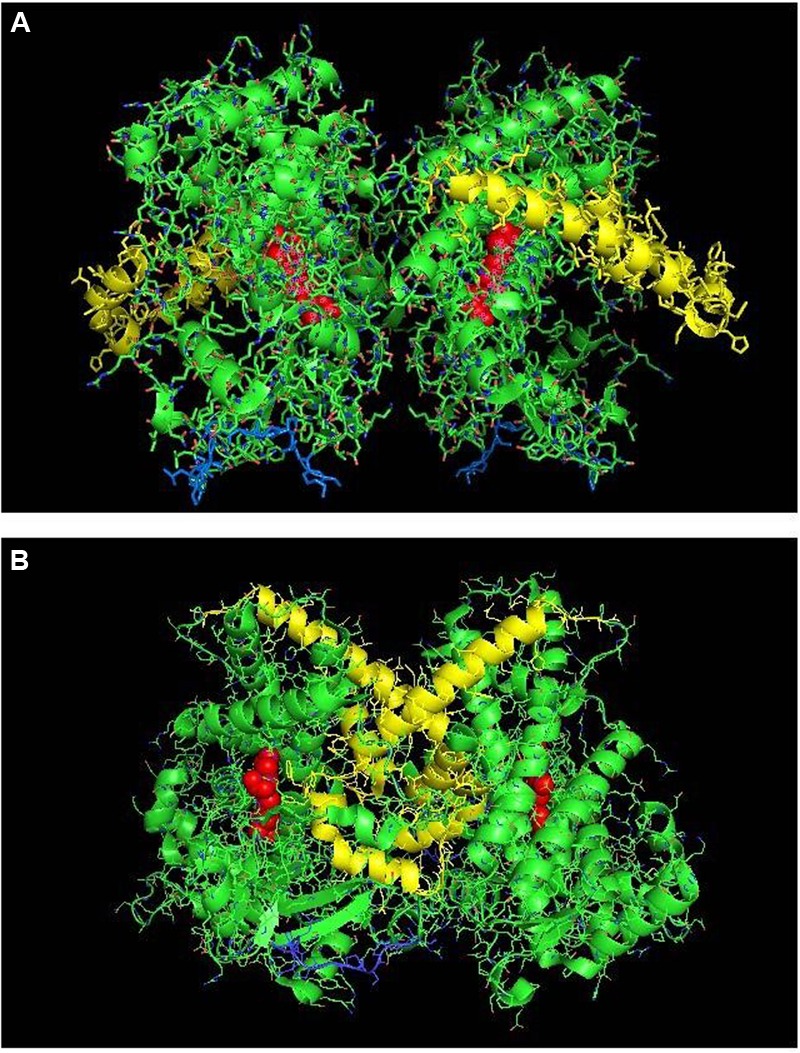
**The crystal structure of open-formed, ligand-free CYP2B4 (PDB:1PO5) showing lattice arrangements of a proximal-to-proximal dimer (A)** and an intercalated, distal-to-distal dimer **(B)**. The units of the dimers are oriented with the N-terminal region (shown in blue) at the base of the dimers in a “membrane plane” running perpendicular to the plane of the image. The heme groups are shown as red spheres with α-helices drawn as cylindrical features. The F-G helices regions on the surface of the distal faces of the enzyme units are shown in yellow.

**FIGURE 6:**
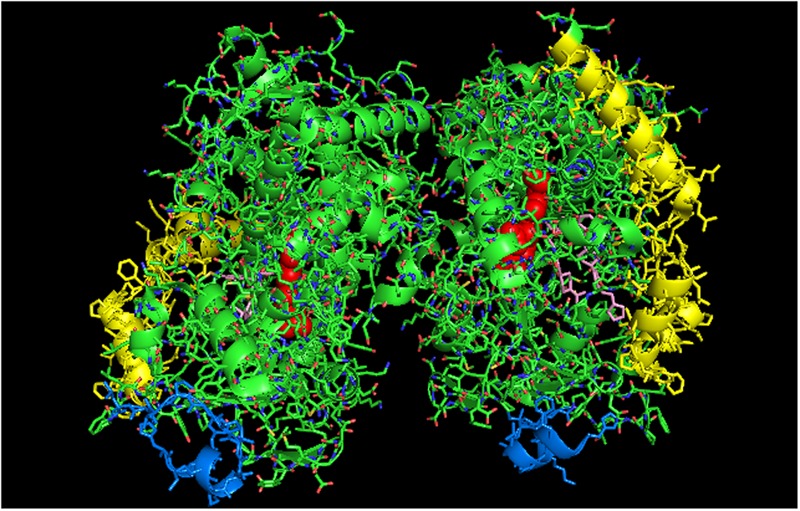
**Crystal structure showing lattice arrangement of proximal-to-proximal dimer of open-formed CYP3A4 with two molecules of ketoconazole bound to each active site (shown in pink) (PDB:2V0M).** The units of the dimer are oriented with the N-terminal region at the base of the dimers in a “membrane plane” running perpendicular to the plane of the image. The heme groups are shown as red spheres with α-helices drawn as cylindrical features. The two ketoconazole molecules bound to each active site are shown in pink.

**FIGURE 7:**
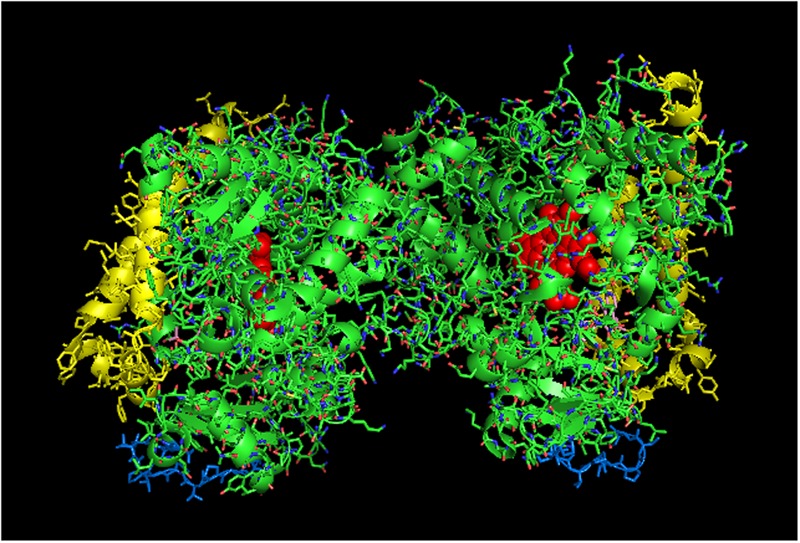
**Crystal structure of side-to-proximal dimer of CYP2C9 bound to warfarin (PDB: 1OG5).** The units of the dimer are oriented with the N-terminal region (shown in blue) at the base of the dimers in a “membrane plane” running perpendicular to the plane of the image. The heme groups are shown as red spheres with α-helices drawn as cylindrical features. The F-G helices region on the distal surfaces of the dimer are shown in yellow. Warfarin molecules (pink) are bound in the active sites.

**FIGURE 8:**
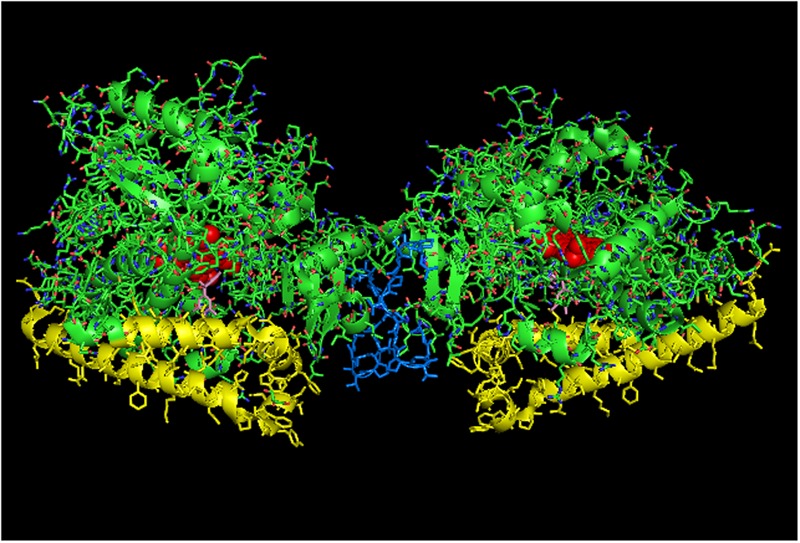
**Crystal structure of side-to-side dimer of CYP2C19 bound to (2-methyl-1-benzofuran-3-yl)-(4-hydroxy-3,5-dimethylphenyl) methanon (PDB: 4GQS).** This arrangement typifies the interactions that can be observed in the CYP2C9 crystal lattice. The units of the dimer are oriented with the N-terminal region at the base of the dimers in a “membrane plane” running perpendicular to the plane of the image. The crystal is predominated by many side-on interactions, many of which do not orient in a manner consistent with being incorporated in a single membrane bilayer. The heme groups are shown as red spheres with α-helices drawn as cylindrical features. The molecules of (2-methyl-1-benzofuran-3-yl)-(4-hydroxy-3, 5-dimethylphenyl) methanon bound to the active site are shown in pink.

**FIGURE 9:**
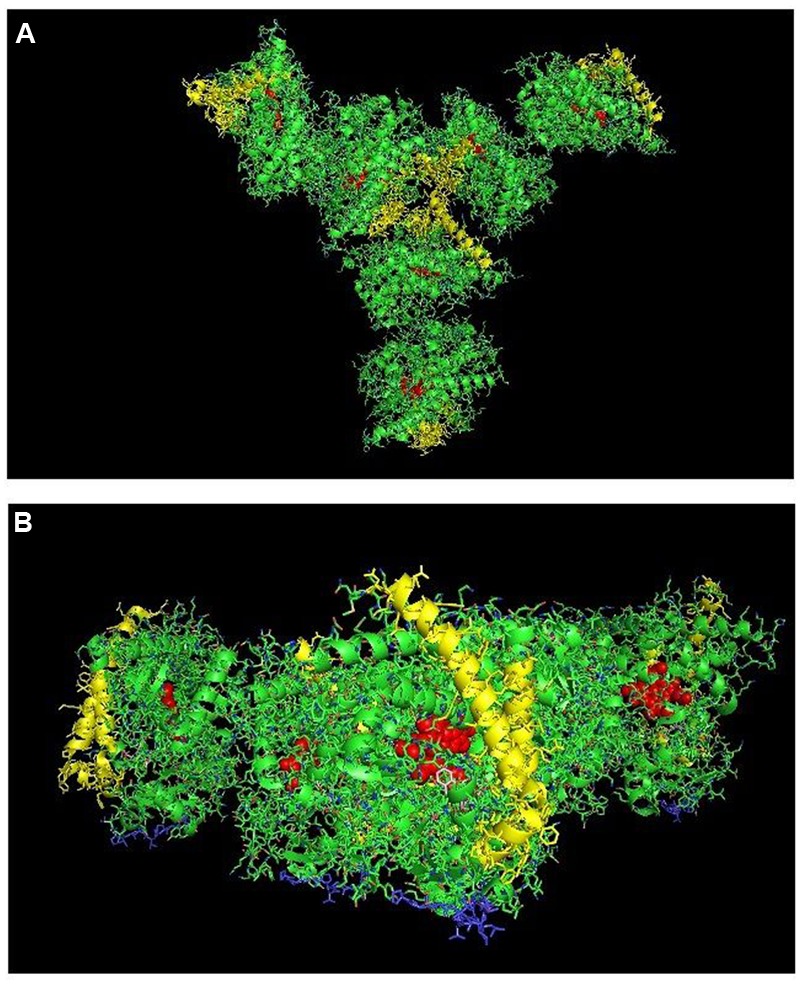
**Crystal lattice of side-to-proximal dimer of CYP2C9 (PDB: 1OG5) (shown in **Figure 7**) showing conceivable hexameric association within a membrane.** The crystal lattice of the side-to-proximal dimer shown in **Figure 7** was examined for higher ordered quaternary structure. **(A)** Shows a trimeric association of the dimers in threefold symmetry formed from a side abutting in an angle against a neighboring distal region of an adjacent CYP2C9 unit. The N-terminal, membrane-binding region for each unit is at the bottom of the hexamer in the plane of the page. **(B)** Shows the same hexamer with the blue N-terminal ends arranged at the bottom of the hexamer in a common plane that runs perpendicular to the plane of the image. The heme groups are shown as red spheres with α-helices drawn as cylindrical features. The F-G helices region on the distal faces of the hexamer units are shown in yellow.

*The binding characteristics of P450 homomeric complexes shown in **Figures 1–9** were determined by using the program PDBePISA (EMBL-EBI, Hinxton, England). ^a^*N*_res_ – The number of interacting residues in the complex. ^b^**Δ**^i^G – the solvation free energy gain upon formation of the interface of the P450–P450 complex. ^c^*N*_HB_ – The number of hydrogen bonds detected. ^d^*N*_SB_ – The number of salt bridges detected. ^e^*N*_DS_ – The number of disulfide bonds detected. ^f^CSS – Complexation significance score. ^g^**Figure 4** depicts a trimer of CYP2B4. Interface 1 corresponds to the interaction of the center unit with the left-most unit of CYP2B4, whereas interaction 2 corresponds to the right-most interaction. ^h^The figure represents a hexamer of CYP2C9. The interactions comprising the “arms” of the hexamer are those depicted in **Figure 7**. Thus, the values shown for **Figure 9** represent those for the central interacting units of the hexamer.*

Examination of the available crystal lattices of P450s shows many different types of plausible interactions that could exist in the membrane including distal-to-distal, proximal-to-proximal, and those involving the sides between the proximal and distal faces of the enzymes. Interactions involving the sides between distal and proximal faces of a P450 will be referred to as side-on or side-to-side interactions. These side-on interactions also can involve contacts between the proximal and distal regions of the interacting partner. Direct interactions between the proximal and distal sides are not commonly observed in the crystal structures for mammalian P450s. However, this arrangement has been identified in the crystal structure of the bacterial reductase-P450 fusion protein, BM3 (Ravichandran et al., 1993). Our analysis will start with examples of crystal structures that have been demonstrated to be likely representations of the homomeric interactions of certain P450s in membranes.

### Homomeric Crystal Structures That have been Supported by Data in Cellular Systems

#### Homomeric CYP2C8

Computer modeling and hydropathy analyses indicate that lipophilic P450 substrates presumably enter the active site via the B-C loop and F-G helices region in the membrane, and the P450-generated products exit through a solvent access channel often found between the F and I helices (Petrek et al., 2006, 2007; Cojocaru et al., 2011). Thus, the distal region plays an important role in substrate binding to most mammalian P450s. A distal-to-distal arrangement in P450 crystal structures has been observed with many different drug-metabolizing P450s including CYP3A4 (1W0F), CYP1A2 (PDB: 2HI4), CYP2D6 (PDB: 2F9Q), CYP2A6 (PDB: 2FDV), and the open form of CYP2B4 (PDB: 1PO5). Recent studies involving CYP2C8 (PDB:1PQ2) (Hu et al., 2010) and CYP3A4 (Davydov et al., 2013, 2015) have provided evidence that this type of dimer is also formed from the homomeric interactions of these P450s when they are membrane-bound.

Studies with purified CYP2C8 in which Trp residues were introduced by mutagenesis in order to examine the interaction (by Trp fluorescence quenching) of the mutated P450 with spin-labeled lipids in artificial membranes (Ozalp et al., 2006) suggested that the F-G loop did not interact with the membrane as predicted from similar studies with human CYP2J2 (McDougle et al., 2015) and hydropathy analysis of a similar CYP2C enzyme (Williams et al., 2000b). Interestingly, this region also was the major point of interaction in the dimeric X-ray crystal structure of CYP2C8 (PDB:1PQ2; **Figure 1**; **Table 1**). It was predicted from the crystal structure that the dimerization would be facilitated by adjacent sulfhydryl groups in cysteine residues of the interacting CYP2C8 units. Dimerization was then demonstrated on electrophoretic gels after either oxidation or cross-linking of the cysteine residues on CYP2C8 that was transiently expressed in bacterial membranes and in AD-293 cells (Hu et al., 2010). Subsequent mutagenesis experiments indicated that dimerization through the sulfhydryls was dependent on having cysteine residues within either the polar linker sequence (between the N-terminal, membrane-spanning region and the catalytic domain) or the F-G loop. Thus, these regions were implicated as the points of contact in the membrane-bound CYP2C8 dimer.

Dimerization through the sulfhydryls was not observed in the cell membranes when the hydrophobic N-terminal membrane anchor was deleted or substituted with a hydrophilic N-terminal sequence. This is consistent with BiFC (Ozalp et al., 2005) and FRET (Szczesna-Skorupa et al., 2003) studies of the closely related CYP2C2 in COS1 cells (discussed above) showing that homomeric P450–P450 interactions required the N-terminal membrane domain. Similarly, N-terminal truncation prevented the interaction of CYP3A4 and CYP2C9 (Subramanian et al., 2010). Thus, the N-terminal region is very important in stabilizing some P450–P450 interactions in the membrane.

Interestingly, the arrangement of subunits in the crystal structure of CYP2C8 were such that the cysteines in the polar linker regions of adjacent units did not align in close enough proximity to allow for disulfides and/or cross linking to occur between the units. In fact, for the polar-linking regions of the dimer units to be in close proximity, the F-G loop would have to be pulled out of the membrane. Thus, dimers with similar features were apparently formed with both full-length CYP2C8 in membranes and with truncated CYP2C8 in solution. However, the membrane interaction that is stabilized by the N-terminal sequence of the full-length form imparts a slight change in alignment within the dimer that allows for cross-linking through adjacent sulfhydryls in the polar linker region. It was speculated that the high enzyme concentrations needed to form CYP2C8 crystals facilitated dimerization even without the N-terminal sequences. However, the N-terminal segment was needed for stabilization of the dimer after transient expression of the enzyme in cellular membranes. Unfortunately, the functional effects of the CYP2C8 homomeric interaction were not assessed. However, as discussed above, movement of the F-G loops, which form part of the substrate access channel from out of the membrane bilayer upon the formation of the dimer, would undoubtedly influence the binding of substrate by the enzyme.

#### Homomeric CYP3A4 and Its Functional Effects

CYP3A4 is quantitatively the most important P450 in drug metabolism (Anzenbacher and Anzenbacherová, 2001; Guengerich, 2003). As a result, considerable effort has been devoted to resolving crystal structures for this enzyme, and a number of conformations have been identified for the P450 when bound to different compounds (Williams et al., 2004; Yano et al., 2004; Ekroos and Sjogren, 2006). Several lines of evidence have suggested that a dimeric arrangement of CYP3A4 in the crystal lattice has functional relevance to the multimeric state of the membrane-bound enzyme. The quaternary structure is unusual because it has two molecules of progesterone bound to a peripheral “cleft” formed in the interface of a homomeric dimer [PDB: 1W0F; **Figure 2**)]. Interestingly, the CYP2C8 crystal dimer (PDB: 1PQ2) also displayed a peripheral binding site for two palmitate molecules in a similar location to that of the CYP3A4 dimer, but its functional significance was not explored.

Davydov et al. (2016) have now provided evidence from studies using LRET/FRET and chemical cross-linking that the CYP3A4 crystal dimer is a reasonable analog to a quaternary structure adopted in the membrane. First, it was described above how FRET/LRET studies indicated that the binding of ANF to CYP3A4 could change the oligomeric state of the enzyme. FRET was also measured after mixing different combinations of specific cysteine-depleted mutants that were variably labeled with donor/acceptor FRET pairs (Tsalkova et al., 2007; Sineva et al., 2013). From dramatic FRET changes upon the binding of the effector, ANF, it was possible to prove a change in conformation that was not elicited when a prototypical substrate bound to the heme active site. These findings are consistent with the hyperbaric study (described above) monitoring a pressure-dependent conformation change in CYP3A4 that was facilitated by the allosteric effector, testosterone (Davydov et al., 2016).

Using another approach, the FRET/LRET efficiencies and the Fṏrster radius of the donor/acceptor pair, it was possible to approximate the separation distance between adjacent cysteines on an interacting pair of variably labeled CYP3A4 cysteine mutants (Davydov et al., 2015). For each pair of mutants, the calculated distances between the cysteine locations were within the range of error of the distances that would be expected in the progesterone-bound crystal. Similarly, by using cysteine-reactive cross-linkers of different lengths with the CYS-depleted CYP3A4 mutants in order to explore the distance between adjacent cysteines in CYP3A4 multimers, it was possible to demonstrate that the results were also consistent with the progesterone-bound CYP3A4 structure. From these studies, it was proposed that ANF and other effectors bound to the same peripheral site bound by progesterone in the implicated crystal structure, and that its binding changed the oligomerization state of the enzyme and in turn, its catalytic activity. From the crystal structure, it was proposed that the progesterone-binding site could play a role in initial substrate recognition because it lines up with a suitable route (through the F-G helices region) from the membrane to the active site (Williams et al., 2004). Thus, in light of the other biophysical data, the conformation change required to open up this putative channel may be facilitated by interactions with the peripheral ligand. Interestingly, the less catalytically active form of CYP3A4 in the absence of effector binding may also be a distal-to-distal dimer (from crystal structure PDB:1W0G). Thus, the change in oligomerization caused by effector binding to the peripheral binding may only be a subtle repositioning of the dimer that opens up a putative substrate access channel through the F-G helices region and not necessarily a change from the monomeric state to a multimeric enzyme complex.

Metabolism by CYP3A4 is often characterized by sigmoidal, homo-, and heterotropic cooperativity (Korzekwa et al., 1998; Shou et al., 2001; Houston and Galetin, 2005). One of the best examples of heterotropic cooperativity involves the effects of ANF on CYP3A4-mediated metabolism. This compound often activates metabolism by eliminating sigmoidal enzyme kinetics. Until recently, cooperativity was attributed to a large active site that is capable of binding multiple substrates. Effector compounds could thus influence positioning of a substrate (and in turn its metabolism) by simultaneous binding to the active site. It has also been postulated that effector binding may elicit a change in CYP3A4 conformation that is more catalytically active. In support of these ideas are crystal structures of CYP3A4 (Ekroos and Sjogren, 2006) showing multiple compounds within the active site (PDB: 2V0M) as well as variations in conformation upon binding to different substrates (PDB: 2J0D). Various biophysical techniques also indicate a ligand-influenced equilibrium between multiple conformations of the enzyme (Koley et al., 1995; Sineva et al., 2013; Davydov et al., 2016). In addition, studies with CYP3A4, reconstituted in nanodiscs with excess CPR, observed both homotropically cooperative substrate binding (Baas et al., 2004) and heterotropically cooperative substrate metabolism (Denisov et al., 2015).

In contrast, the LRET/FRET studies with CYP3A4 described above suggest that effector-induced changes in protein conformation lead to changes in protein oligomerization, and it may in fact be the latter changes that directly relate to cooperative metabolism by CYP3A4. The relative roles that effector-mediated substrate re-positioning in the active site and effector-mediated changes in protein conformation/oligomerization play in causing cooperative CYP3A4-mediated metabolism have not yet been determined. However, one of the objectives of this review is to entertain the possibility that many functional changes of P450–P450 interactions could simply be explained by positional changes of enzyme units in P450 multimers and not solely by changes in enzyme conformation and substrate-positioning within the active site.

### Using Positional Re-arrangement within P450 Multimers to Explain the Functional Effects of P450–P450 Interactions

The reported functional effects involving a given P450 that are caused by its interaction with another P450 will be considered with respect to any distinctive features observed in the crystal lattice arrangement of the P450 that may impart functional properties to the homomeric complex. The validity of this approach lies in the fact that any discussion about the functional effects of P450–P450 interactions is relative to the frame of reference. As discussed in detail above, the P450 units in homomeric multimers are not identically distributed within the aggregate but are characterized by different spin states and different abilities to be reduced/bind substrates. Furthermore, the transition between subunit positions within the aggregate is extremely limited or hindered as indicated by removal of a segment of the P450 population from the rapid spin state equilibrium. Thus, any aggregate of P450 is inhibitory by nature of the fact that some of the units in the aggregate may not be fully functional. With this in mind, any heteromeric P450–P450 interaction that causes activation of a P450 could be due to the homomeric interactions of the affected P450 being relatively inhibitory. Conversely, inhibitory P450–P450 interactions would be related to subunit arrangements in the hetero-complex that contrast with those associated with higher functionality in the homomeric complex. Studies that have elucidated the kinetic mechanisms by which P450–P450 interactions influence metabolism will be used to explain how specific homomeric interactions may be more or less functional.

### P450–P450 Interactions That Influence CPR Binding

#### CYP1A2 Interactions

Several studies have demonstrated that the ability of a P450 to bind CPR is altered when it forms a mixed complex with another P450. For the P450–P450 interactions of CYP1A2-CYP2B4 (Backes et al., 1998), CYP1A2-CYP2E1 (Kelley et al., 2006), CYP2C9-CYP2C19 (Hazai and Kupfer, 2005), and CYP2E1-CYP2B4 (Kenaan et al., 2013), CYP1A2 and CYP2C9 (and CYP2E1 in its interaction with CYP2B4) were able to bind CPR more readily (and thus be more catalytically active) when bound to the indicated P450s; whereas the ability of the interacting P450 to bind CPR was either inhibited or unaffected. If one only considers the relative positions of the protein “units” in a P450 multimer (and not necessarily the conformation of the enzyme as a determinant in its activity), CPR binding could most logically be affected by interactions involving the proximal side of the P450 enzyme sterically disturbing formation of the CPR⋅P450 complex and consequently electron transfer.

Thus, taking the CYP1A2-CYP2B4 interaction as the most well-studied example of this effect and the line of reasoning used in the introductory paragraph to this section, the activation of CYP1A2 via enhanced CPR binding in mixed P450–P450 complexes would imply that its homomeric complex is limited in its ability to bind CPR. In support of this conclusion, research from our lab has clearly demonstrated that the homomeric interaction of CYP1A2 inhibits its activity by a mechanism that involves a lower CPR-binding affinity (Reed et al., 2012). In these studies, CYP1A2 activities showed a sigmoidal response as a function of CPR concentration, which is consistent with a CYP1A2⋅CYP1A2 dimer that is inactive (Reed et al., 2012).

Interestingly, a proximal-to-proximal interaction is very prevalent in the crystal lattice for CYP1A2 (PDB: 2HI4, **Figure 3**). With this proximal-to-proximal arrangement, the units in the CYP1A2 dimer directly overlay the proximal face of the identical binding partner in a manner that would completely prevent access for CPR binding. Thus, the stimulation of CYP1A2 caused by heteromeric P450 interactions (with either CYP2B4 or CYP2E1) may stem from the CYP1A2 having a different arrangement with its proximal face exposed in the presence of these other P450s. This in turn, would cause a higher proportion of the CYP1A2 to be available to functionally interact with CPR.

### Proximal-to-Proximal Interactions with Open Conformations of P450s

The proximal-to-proximal interaction is very unusual in closed forms of P450s. For instance, the closed conformation of CYP2B4 (PDB: 1SUO) is predominated by side-to-side interactions (**Figure 4**). However, a proximal-to-proximal interaction is observed in the crystal lattice of an open form of CYP2B4 (PDB:1PO5; **Figure 5A**). The open form of this P450 was crystallized as a distal-to-distal dimer (**Figure 5B**). This conformation of CYP2B4 adopts many different arrangements in its crystal structure including two types of side-to-side interactions in addition to the distal-to-distal and proximal-to-proximal orientations. The open-form, intercalated, distal-to-distal dimer represents an inactive complex having a His in the F-helix of one open unit coordinating with the active site heme of the other open CYP2B4 unit. An evaluation of the open conformation of the CYP2B4 crystal structure led to the conclusion that this form of CYP2B4 also would be deficient in functionally interacting with CPR because of repositioning of the C helix (Scott et al., 2003). Thus, whether or not the open CYP2B4 is in a proximal-to-proximal interaction, it is probably not reducible by CPR. In the inhibition of CYP2B4 caused by its interaction with CYP1A2, it seems conceivable that the effect could be due in part, to a proximal-to-proximal association of open form CYP2B4 with CYP1A2 in which the CYP2B4-binding site for CPR is blocked. The hyperbaric studies of the CYP1A2-CYP2B4 complex (Davydov et al., 2000b) showed that there was increased hydration of the active site of CYP2B4 as a result of its interaction with CYP1A2 which is consistent with the interaction involving the open form of CYP2B4.

A proximal-to-proximal interaction can also be observed in the crystal lattice of an open form of CYP3A4 bound to ketoconazole (PDB: 2V0M; **Figure 6**) (Ekroos and Sjogren, 2006). Furthermore, the crystal structure of the open CYP3A4 dimer associated with the binding of erythromycin is arranged in an offset proximal-to-proximal orientation (PDB: 2J0D). As is the case with CYP2B4, proximal-to-proximal interactions of the open CYP3A4 are not observed in crystal lattices of closed CYP3A4 forms (discussed above). CYP3A4 metabolizes very large substrates, and the open form apparently is adopted when large substrates are metabolized by the P450. Thus, unlike CYP2B4, the open CYP3A4 form is likely to be catalytically active. Similar to the crystal lattice of open CYP2B4, the open form of CYP3A4 also displays a variety of arrangements including distal-to-distal (not intercalated) and side-to-side. Thus, these open forms may be versatile in the types of associations they can adopt.

The open conformation has also been implicated for a R108H mutant of CYP2C9 through the use of size exclusion chromatography, UV-visible spectrophotometry, pulsed electron paramagnetic resonance, and molecular dynamics simulations (Roberts et al., 2010). The mutant was isolated in a conformation in which the substituted His in the B-C loop coordinated *intramolecularly* with the heme group. Thus, this form is a monomer, but the mobility of the B-C loop in coordinating with the heme is similar to the conformational mobility needed to form the open conformation of CYP2B4. Because this conformation was ascertained by physical techniques other than X-ray crystallography, the tendency of this form to associate in a proximal-to-proximal orientation cannot be determined.

Any open form in which inter- or intramolecular rearrangement blocks substrate access to the active site would be inhibitory. Thus, it seems open conformations of P450s may in general, be a means to limit the activities of the P450s by favoring the formation of proximal-to-proximal arrangements that are deficient in binding to CPR. Unfortunately, very few P450 crystal structures have been isolated in the open conformation to confirm that this form consistently adopts these arrangements. Thus, this hypothesis needs further testing. However, it should be pointed out that the arrangement is observed in all of the available open-form crystal structures.

It is clear that the crystal structure of CYP1A2 is unusual in adopting a proximal-to-proximal arrangement in the closed conformation. Furthermore, it can also be argued that CYP1A2 would be incapable of adopting the open conformation given its narrow active site that is conducive for metabolism of planar, aromatic substrates (Sansen et al., 2007). If the conformation of CYP1A2 did open up, the P450 would be expected to metabolize a wider range of substrates. Thus, the proximal-to-proximal interaction may have an important regulatory role in P450-mediated catalysis, and CYP1A2 may utilize its ability to adopt this arrangement in the closed form to influence the conformation and quaternary structure of interacting P450s.

### CYP2C9 Interactions

Stimulation of CYP2C9-mediated activity through a mechanism involving higher CPR binding affinity was also reported for the interaction of the P450 with CYP2C19 (Hazai and Kupfer, 2005). An explanation for this effect was proposed through an examination of CYP2C9 crystal structure in a subsequent study (Hazai et al., 2005). At the time of this study, the number of available crystal structures for mammalian P450s was limited, and the functional effects of P450–P450 interactions were interpreted solely by the potential effects on CPR binding as this was the only identified mechanism.

To explain the activation of CYP2C9 metabolism by its interaction with CYP2C19, the previous study examined a side-to-proximal dimer (PDB: 1OG5; **Figure 7**) which has the proximal face of one unit being occluded by the side of the protein containing the C-terminal end of the I-helix and the J-helix. In the previous study, it was postulated that the CYP2C9 dimer would bind CPR with greater affinity than a CYP2C9 monomer because the exposed J and K helices of the obstructed proximal face would contribute to CPR binding by the exposed proximal face of the self-interacting CYP2C9 unit. Although this is an intriguing conclusion to be drawn from this crystal structure arrangement, it must also be considered that this complex instead represents an inhibitory, homomeric CYP2C9 complex because one of the two proximal faces is sterically hindered in its ability to bind CPR. Thus, at least one of the two CYP2C9 units in the dimer would be catalytically limited. Furthermore, it is possible that CPR binding to the “exposed” proximal side of the alternate unit also would be limited because it is partially obstructed in the crystal structure.

Because positional heterogeneity implies that any heteromeric P450–P450 interaction stimulating a P450 catalytic activity can also be interpreted as the homomeric interactions being inhibitory, the side-to-proximal interaction of CYP2C9 may instead be inhibitory and not one that is characterized by increased CPR-binding affinity. As a result, the stimulation of CYP2C9 through its interaction with CYP2C19 may be mediated through a quaternary structure that fully exposes the CYP2C9 proximal face, possibly doubling the amount of catalytically active CYP2C9 relative to what would be observed with the homomeric CYP2C9 dimer (PDB: 1OG5). The CYP2C19 crystal lattice (PDB:4GQS; **Figure 8**) shows one arrangement that would be a credible membrane alignment in which the N-terminal ends of a symmetrical dimer interact in a manner that would expose the proximal faces at a rather flat angle to the membrane normal with the distal faces buried below the membrane surface. In conjunction with this arrangement, many hydrophobic residues on the distal side project below what would be the membrane surface in this orientation. Thus, the functional effects reported for this interaction could be explained if a greater proportion of CYP2C9 adopted the “CYP2C19-like” arrangement at the expense of the side-to-proximal dimer associated with the CYP2C9 crystal lattice, and more of the CYP2C19 adopted the CYP2C9 homodimer arrangement.

The distinction between a conformational mechanism and the one involving a change in the arrangement of a P450 complex that exposes the proximal face of the P450 is significant. In the second case, the amount of a specific P450 participating in the metabolism of a substrate would increase because a higher proportion of the P450 would have the ability to interact with CPR. It is important to note that this effect could mistakenly be attributed to a change in the rate of a catalytic step of the reaction cycle. To take the effects of the CYP2C9-CYP2C19 interaction as an example (Hazai and Kupfer, 2005), stimulation of the CYP2C9-mediated metabolism of methoxychlor was observed in the presence of CYP2C19 when CPR was at a saturating concentration. This would not happen if the change was caused by a conformation-related increase in the binding affinity of CPR and CYP2C9 because the CPR level was saturating both in the presence and absence of CYP2C19. However, the effect could be explained if the interaction of CYP2C9 with CYP2C19 causes more of the proximal face of CYP2C9 to be exposed for binding to CPR. Thus, any P450–P450 interaction that has been attributed to a change in the rate of a catalytic step, other than substrate binding, may in fact be caused by this mechanism instead. In other words, any apparent change in the *k*_cat_ of a reaction associated with a P450–P450 interaction could instead be attributable to an alteration in the amount of available active enzyme through a ***re-***rearrangement within a P450 multimer that exposes or hides more of the proximal face of the affected P450 (*i.e., an effective increase it total active enzyme would present as an increase in k_cat_*).

Another important kinetic distinction in the formation of the proximal-to-proximal dimer is that the P450 in this arrangement would be effectively removed from the population of P450 capable of binding CPR. Thus, if there is a net exchange of one P450 form for another in this arrangement, the effective CPR:P450 ratio would increase for the P450 that is activated through this mechanism. This would be another potential mechanism for enzymatic stimulation in the mixed system.

### P450–P450 Interactions That Influence Substrate Binding

As reviewed previously (Reed and Backes, 2016), the interactions of CYP2C9 with CYP2D6 (Subramanian et al., 2009) (and possibly with CYP3A4) (Subramanian et al., 2010) and CYP2B4 with CYP2E1 (Kenaan et al., 2013) have been shown to cause inhibition of CYP2C9 and CYP2B4, respectively, by increasing the dissociation constants for substrate binding. Changes in substrate binding could be plausibly explained through P450–P450 interactions that involve the distal side of the affected P450. A distal-to-distal P450 quaternary structure is commonly observed in P450 crystal lattices. A membrane-bound analog of this type of dimer is typified by the crystal structure of CYP2C8 (Hu et al., 2010). In these types of dimers, the P450s can assume either closed or open conformations around the active site, and the F and G helices of the interacting P450s form the dimerization interface. Interestingly, the dimerization of CYP2C8 probably causes the F/G loops to be pulled out of the membrane bilayer (Schoch et al., 2004; Ozalp et al., 2006). This effect in turn, would likely affect substrate binding to the enzyme and would probably inhibit metabolism of hydrophobic substrates that partition largely to the membrane. Perhaps, in its interaction with CYP2C9, CYP2D6 may favor a rearrangement of the CYP2C9 that obstructs its distal face and in turn, causes its substrate to bind less tightly to its active site and inhibits CYP2C9-mediated metabolism in the process. Similarly, by consideration of positional heterogeneity as the primary determinant of P450–P450 effects, it would be presumed that CYP3A4 binds to the distal side of CYP2C9 to disrupt binding of S-naproxen and *S*-flurbiprofen, and in a similar manner, CYP2E1 (at high enzyme concentrations) distorts the distal side of CYP2B4 and negatively influences binding of benzphetamine to its active site (Kenaan et al., 2013).

A more recent study (Bostick et al., 2016) has reported that the interaction of CYP2D6 and CYP2C9 resulted in stimulation of the CYP2C9-mediated metabolism of *S*-flurbiprofen [the same substrate in the study reporting inhibition of CYP2C9 (Subramanian et al., 2009)]. There were several distinctions in the methods used in the two studies that could explain the discrepancy in the findings. Most importantly, the reaction went for 16 h in the later study, and it was reported that the rates were not linear over this time period. Thus, “stimulation” could merely reflect longer enzymatic stability when CYP2C9 was in the mixed complex, and the earlier study probably describes the effects of the CYP2C9-CYP2D6 interaction more accurately.

### Putative Hexameric Quaternary Structure of Membrane-Bound CYP2C9

In examining crystal lattices of purified P450s for the putative hexameric aggregate that has been implicated by electron microscopy (Tsuprun et al., 1986; Myasoedova and Tsuprun, 1993) and saturation transfer electron paramagnetic resonance (Schwarz et al., 1982), an interesting arrangement can be observed in the crystal lattice of CYP2C9 (PDB: 1OG5; **Figures 9A,B**) in which a large-scale pattern that includes a trimer of asymmetric dimers can be observed. The dimers referred to are the side-to-proximal dimers described above. The arrangement is intriguing in that it is consistent with functional membrane binding and has similar features to the hexameric structures of CYP1A2 (Myasoedova and Tsuprun, 1993) and CYP2B4 (Tsuprun et al., 1986) observed by electron microscopy as it is comprised of two types of contacts between the CYP2C9 units of the hexamer. In addition to the side-to-proximal interaction, the three sets of dimers are joined together with threefold symmetry along the distal faces and the side between the proximal and distal faces that contains the regions between the B/C helices and the H/I helices in addition to the C-terminus of the enzyme. Future research should consider this hexamer as a possible quaternary structure when metabolism by CYP2C9 is assessed in the context of P450–P450 interactions.

## Discussion

Physical studies have convincingly shown that P450s have a propensity to form complexes in membranes. Studies measuring FRET/LRET and surface plasmon resonance now allow for determinations of the dynamics of P450–P450 association. Measurements indicate dissociation constants ranging from 1 to 50 nM (0.05–0.6 pmol/m^2^ for calculations based on the two-dimensional lipid bilayer). Thus, P450s bind with affinities that are higher than those measured for some signaling complexes within the plasma membrane where the dissociation constants of various *G*_α_ subunits for the *G*_βγ_ subunit, coupled to the *N*-formyl peptide receptor, ranged from 1 to 3 μM (Bennett et al., 2001).

Studies with cross-linkers and those designed to monitor the rotational mobility of P450s indicate the presence of dimeric to hexameric (and larger?) P450 multimers in membranes with some P450–P450 interactions being governed by electrostatic forces possibly involving amine groups in the polar linker region and/or the proximal faces of the enzymes. In natural membranes, P450s form complexes of varying sizes with higher ordered aggregates containing essentially rotationally immobilized P450 units.

These P450–P450 interactions would not be of particular interest if they did not impart functional changes that affect drug metabolism. However, the P450 units in these multimers display positional heterogeneity with respect to chemical/enzymatic reduction, spin state, and access to substrate. The heterogeneity of P450 multimers with respect to these properties is a testament to the quasi-irreversible nature of P450–P450 associations and is the underlying source of the functional effects of P450–P450 interactions with regards to drug metabolism.

An integral question concerning the functional effects of P450–P450 interactions involves the physiologic rationale for their existence. Given their important role in the elimination of hydrophobic xenobiotics, it may seem counter-intuitive that their activities could be selectively inhibited by oligomerization. Enzymatic regulation through oligomerization also provides a means for the P450 system to respond immediately to the presence of a given substrate through quaternary re-arrangements that “unmask” otherwise latent P450-mediated activities. If this were not the case, the P450 system might be ineffective at removing the compound until delayed induction of the relevant P450s. In addition, the P450 catalytic cycle depends on the activation of molecular oxygen through the transfer of electrons to the heme group. This process can result in uncoupling and the generation of destructive reactive oxygen species. Thus, the regulation of P450-mediated activities through oligomerization may provide a mechanism to limit deleterious uncoupling reactions. This topic has been addressed more fully in other reviews (Zangar et al., 2004; Davydov, 2011). It is also possible that P450 oligomerization has a more complex role with the expression of specific P450s serving to silence or enhance the activities of P450s with which they tend to interact.

Despite the fact that general mechanisms involving altered CPR and/or substrate binding have been identified for the functional effects of P450–P450 interactions, the specific ways that the interactions cause these changes have been difficult to interpret. Typically, general explanations attribute the phenomena to protein conformation changes. Undoubtedly, undefined conformation changes are the underlying causes given that the effects of P450–P450 interactions are substrate-specific. For instance, all interactions involving CYP2E1 and CYP2B4 do not cause activation of the former and inhibition of the latter (Kelley et al., 2006). However, *p*-nitrophenol and benzphetamine trigger specific conformation changes in CYP2E1 and CYP2B4, respectively, that lead to those effects when the two P450s interact (Kenaan et al., 2013).

While the underlying protein conformational changes that affect oligomerization are important to elucidate, the actual changes in oligomerization affecting positional heterogeneity are possibly the ultimate source of functional changes. In fact, it can be reasoned that certain arrangements of P450s would have very predictable consequences on drug metabolism. Thus, for this review, we have examined the crystal lattices of several P450 structures to identify arrangements that may be analogous to their organization in membranes, with the ultimate objective of identifying potential changes in oligomerization that would explain the alterations in function observed with specific P450–P450 interactions.

To explain the role of oligomerization in affecting the enzymatic function of drug-metabolizing P450s, it is proposed that the degree and the variability of P450 oligomerization represents a P450-specific equilibrium that is influenced by the conformational state of the protein. The conformational variability of most P450s (except CYP1A) is proposed to be distinguished by the open and closed states represented by CYP2B4 crystal structures (PDB: 1PO5 and PDB: 1SUO, respectively), with ligand-free CYP2B4 existing in the membrane in both states. In most cases, substrate binds to or triggers formation of the closed state. The exception to this trend would be cases such as that with CYP3A4 when large substrates are bound to the active site which in itself induces the open conformation of the enzyme.

The model maintains that each P450 displays a specific tendency to distribute to different proportions of P450–P450 associations that can be categorized as distal-to-distal, proximal-to-proximal, side-to-side, and side to either proximal or distal faces. Thus, interactions between different P450s cause a redistribution of each P450 to different proportions of each arrangement which is influenced by the tendency of the binding partner to adopt a specific arrangement. It is proposed that a functional effect is associated with a P450–P450 interaction when the interaction causes changes in the relative proportions (as compared to the proportions observed in homomeric reconstituted systems) of side-on; distal-to-distal; and proximal-to-proximal P450 arrangements. More specifically, an interaction with a P450 that favors distal-to-distal (e.g., CYP2D6, PDB: 2F9Q) or proximal-to-proximal (e.g., CYP1A2) arrangements would be expected to result in inhibition of P450s that participate in predominantly side-on interactions [e.g., CYP2B4 (**Figure 4**) and CYP2C19 (**Figure 8**)]. In conjunction, this has been shown to be the case for several P450–P450 interactions involving these P450s (Hazai and Kupfer, 2005; Reed et al., 2010; Kenaan et al., 2013).

This is not meant to imply that CYP2B4 and CYP2C19 only arrange in side-on interactions in the membrane. Clearly, the functional heterogeneity of CYP2B4 has been demonstrated (Davydov et al., 1985, 1995; Backes and Eyer, 1989) and may represent a mixture of distal-to-distal (and possibly proximal-to-proximal of the open form) in addition to the side-on interactions. Conformational changes caused by substrate binding would be predicted to alter the proportions of the P450 in each characteristic state of oligomerization. Interestingly, distal-to-proximal interactions were not observed in any of the mammalian structures although they have been reported for bacterial P450s (Ravichandran et al., 1993). This leads to the possibility that the arrangement of P450 units in multimers is not random. Thus, distal-to-distal and proximal-to-proximal interactions may be specific mechanisms that have evolved to regulate the activities of membrane-bound P450s.

For most P450s except CYP1A2, the open conformation of P450 appears to be uniquely associated with both proximal-to-proximal and the distal-to-distal dimeric interaction in which the F-helix of one binding partner intercalates with the active site of the interacting P450 (for simplicity, this will be referred to as the intercalated open-form dimer). As described above, both of these open arrangements are inactive. CYP1A2 presents an unusual situation for the drug-metabolizing P450s because it is not thought to be able to adopt the open conformation. However, unlike most of the drug-metabolizing P450s, CYP1A2 can adopt the proximal-to-proximal arrangement when it is in the closed, substrate-binding conformation. For other drug metabolizing P450s, the proximal-to-proximal interaction has only been associated with crystal lattices of open form P450s. Thus, rearrangement of CYP1A2 from its proximal-to-proximal orientation to one associated with optimal catalytic activity upon its interaction with another P450 would stimulate CYP1A2 by increasing the total amount of “enzymatically active CYP1A2” and also by increasing the effective CPR:CYP1A2 ratio. Conversely, the P450 interacting with CYP1A2 may be inhibited because of the increased accessibility of CYP1A2 to the limiting concentrations of CPR. Inhibition may also occur if the affected P450 interacts as an open form in a proximal-to-proximal manner with CYP1A2.

Distal-to-distal interactions commonly observed in many P450 crystal structures in which the units of the P450 multimer interact on the periphery of the distal regions of P450s in the *closed* conformation are proposed to be impaired in substrate binding. The analogous membrane-bound CYP2C8 dimer has been proposed to be deficient in lipophilic substrate binding from the membrane (Hu et al., 2010). Changes in the relative amounts of this type of dimer may explain the functional interactions of CYP2C9-CYP2D6 (Subramanian et al., 2009); CYP2C9-CYP3A4 (Subramanian et al., 2010); and possibly CYP2E1-CYP2B4 (when a high concentration of CYP2E1 interacts with CYP2B4) (Kenaan et al., 2013). CYP inhibition in these instances is caused by an increase in the proportion of the affected P450 in the closed-form, distal-to-distal arrangement and a corresponding decrease in the proportion of enzyme involved in side-on interactions. Homotropic and heterotropic cooperation in metabolism by CYP3A4 have been related to this type of distal-to-distal dimer (Davydov et al., 2013) indicating a limitation in the ability of it to bind substrate until a putative access channel is opened by effector binding to a peripheral site on the adjoining distal regions of the dimer (Williams et al., 2004; Davydov et al., 2015).

The proposed equilibria actually can explain all of the reported functional effects of these interactions. For instance, changes in the proportion of a P450 in a quasi-irreversible proximal-to-proximal arrangement which are caused by interactions with another P450 can explain the functional effects involving modifications in CPR-binding capability. This also includes mechanisms implicating a change in the apparent *k*_cat_ as the positional change of the affected P450 would present more or less of the total enzyme population for interactions with CPR. Note, the heterocomplex would need to be stable in the presence of excess CPR like the CYP2C9-CYP2C19 complex in order for rigorous kinetic analysis to detect an apparent change in *k*_cat_. The purported effects for the proximal-to-proximal interaction are believed to explain the reported functional interactions for homomeric CYP1A2 (Reed et al., 2012) and for those between CYP2C9-CYP2C19 (Hazai and Kupfer, 2005); CYP1A2-CYP2B4 (Backes et al., 1998); and CYP2E1-CYP2B4 (Kenaan et al., 2013).

In Fernando et al. (2008) three functional populations were identified in the study of the positional heterogeneity of P450–P450 complexes. One population responded to substrate by shifting rapidly to the high spin form and subsequently being reduced enzymatically to form the ferrous-CO complex. This could represent closed-form, side-on P450–P450 interactions fully capable of substrate and reductase binding (“HS+” in the Fernando study).

Another population did not shift to high spin upon addition of substrate and could not be reduced (“HS-” in previous study). This characteristic could actually be representative of the intercalated, open-formed P450 dimer and/or the open-form proximal-to-proximal dimer. Clearly, the proximal-to-proximal dimer would not be reducible given the fact that the dimer is physically unable to interact with CPR. Furthermore, if the dimerization limited conversion to the closed conformation, the proximal-to-proximal CYP2B4 dimer also would be expected to be intransigent to a substrate-induced spin state transition. The “HS-” population also could represent the open-form, intercalated dimer of CYP2B4 given the fact that it was thought to be incapable of *functionally* interacting with CPR (note, a putative opened-form dimer of CYP3A4 must be able to interact with CPR in order to metabolize large substrates) and substrate would not bind to the intercalated P450 dimer since the active site is blocked by the F-helix of the binding partner. In support of the possibility that the HS- population is represented (at least in part) by the intercalated dimer, the biphasicity of the pre-steady state reduction of CYP2B4 by CPR was explained by a model involving a slow equilibrium between two conformations that bind to CPR, but only one of which could be reduced (Backes and Eyer, 1989). Thus, these two groups of CYP2B4 would be consistent with P450 in side-on interactions and the open intercalated dimer of CYP2B4. It is important to note that both types of open-form dimerization may be a means to regulate P450 activities and may be related by a dynamic equilibrium. However, because of the constraints of the lipid membrane and the limitation imposed by the P450s being tethered by the amino terminal tail, trimeric quaternary structures comprised of both proximal-to-proximal and distal-to-distal arrangements do not appear to be possible. Thus, the two types of dimers are exclusive of one another.

The final population in the previous study consisted of low spin CYP3A4 that was slowly converted to high spin prior to enzymatic reduction (“HS_0_”). Substrate binding was proposed to slowly shift the enzyme to the high spin/reducible form. This is fully consistent with our predicted properties of the closed-form distal-to-distal P450 complex as substrate binding is proposed to be limited for this population which would in turn, cause a slower spin state transition and subsequent reduction by the reductase. The Fernando study indicated that ANF (the allosteric activator of CYP3A4 that is believed to facilitate substrate binding by interaction at the peripheral effector site of the dimer) was shown to dramatically decrease the proportion of the HS_0_ population. This would be expected if the peripheral binding by ANF facilitates substrate access to the active sites of these closed form CYP3A4 distal-to-distal dimers.

Although there has been significant progress on the topic of the functional effects of P450–P450 interactions, there is still much more that is unknown than known about how these interactions influence catalytic activities and drug metabolism *in vivo*. For instance, of the roughly 40 possible binary P450–P450 interactions in liver that involve the major P450s in drug metabolism and of the approximately 1500 drugs that have been approved by the FDA (Kinch et al., 2014), only about 12 specific P450–P450 interactions related to the metabolism of approximately 30 substrates (many of which are not even drugs) have been studied (Reed and Backes, 2016). Another topic of relevance that needs to be explored is the study of tissue-specific P450–P450 interactions involving the unique assemblages of P450s expressed in each tissue and the consequent functional effects of these interactions.

In total, these findings suggest that heteromeric P450 association serves as a means to regulate specific P450-mediated drug metabolism *in vivo*. Only rigorous comparative studies will determine whether specific P450s preferentially interact. It is possible that all P450s have similar tendencies to interact with one another. If this is the case, identification of the P450s involved in the metabolism of specific drugs (information that is routinely obtained before phase I drug studies) will allow for the determination of specific P450–P450 interactions that influence metabolism of the given drug by the implicated P450. This information will be invaluable in pharmaceutical risk assessment and in pharmacokinetic evaluations of drug metabolism and disposition.

## Author Contributions

Substantial contributions to the conception or design of the work; or the acquisition, analysis, or interpretation of data for the work – JR and WB. Drafting the work or revising it critically for important intellectual content – JR and WB. Final approval of the version to be published – JR and WB. Agreement to be accountable for all aspects of the work in ensuring that questions related to the accuracy or integrity of any part of the work are appropriately investigated and resolved – JR and WB.

## Conflict of Interest Statement

The authors declare that the research was conducted in the absence of any commercial or financial relationships that could be construed as a potential conflict of interest.
